# Differential effects of *PCSK9* variants on risk of coronary disease and ischaemic stroke

**DOI:** 10.1093/eurheartj/ehx373

**Published:** 2017-07-17

**Authors:** Jemma C Hopewell, Rainer Malik, Elsa Valdés-Márquez, Bradford B Worrall, Rory Collins, METASTROKE Collaboration of the ISGC

**Affiliations:** 1 Clinical Trial Service Unit and Epidemiological Studies Unit, Nuffield Department of Population Health, University of Oxford, BHF Centre for Research Excellence, Big Data Institute, Old Road Campus, Roosevelt Drive, Oxford OX3 7LF, UK; 2 Institute for Stroke and Dementia Research, Klinikum der Universität München, Ludwig-Maximilians-University, Feodor-Lynen-Straße 17, 81377 Munich, Germany; 3 Department of Neurology, University of Virginia Health System, McKim Hall, Hospital Drive, Charlottesville, VA 22908, USA

**Keywords:** PCSK9, Genetics, LDL-cholesterol, Coronary disease, Stroke, Cardiovascular therapies

## Abstract

**Aims:**

*PCSK9* genetic variants that have large effects on low-density lipoprotein cholesterol (LDL-C) and coronary heart disease (CHD) have prompted the development of therapeutic PCSK9-inhibition. However, there is limited evidence that *PCSK9* variants are associated with ischaemic stroke (IS).

**Methods and results:**

Associations of the loss-of-function *PCSK9* genetic variant (rs11591147; R46L), and five additional *PCSK9* variants, with IS and IS subtypes (cardioembolic, large vessel, and small vessel) were estimated in a meta-analysis involving 10 307 IS cases and 19 326 controls of European ancestry. They were then compared with the associations of these variants with LDL-C levels (in up to 172 970 individuals) and CHD (in up to 60 801 CHD cases and 123 504 controls). The rs11591147 T allele was associated with 0.5 mmol/L lower LDL-C level (*P* *=* 9 × 10^−143^) and 23% lower CHD risk [odds ratio (OR): 0.77, 95% confidence interval (CI): 0.69*–*0.87, *P* *=* 7 × 10^−6^]. However, it was not associated with risk of IS (OR: 1.04, 95% CI: 0.84*–*1.28, *P* *=* 0.74) or IS subtypes. Information from additional *PCSK9* variants also indicated consistently weaker effects on IS than on CHD.

**Conclusion:**

*PCSK9* genetic variants that confer life-long lower PCSK9 and LDL-C levels appear to have significantly weaker, if any, associations with risk of IS than with risk of CHD. By contrast, similar proportional reductions in risks of IS and CHD have been observed in randomized trials of therapeutic PCSK9-inhibition. These findings have implications for our understanding of when Mendelian randomization can be relied upon to predict the effects of therapeutic interventions.

## Introduction

The causal association between low-density lipoprotein cholesterol (LDL-C) level and coronary heart disease (CHD) risk is well established. However, the observational and randomized evidence for the associations of LDL-C with ischaemic stroke (IS) risk is contrasting. In observational studies, LDL-C is much more weakly associated with IS than with CHD (about 10% vs. 30% lower relative risk per 1 mmol/L lower LDL-C).^1^ By contrast, in randomized controlled trials, statin therapy that lowered LDL-C levels for about 5 years has been found to produce similar proportional reductions in the risks of IS and CHD (20–25% per 1 mmol/L LDL-C reduction).^2^ Ezetimibe, which lowers LDL-C by a different mechanism to statins, also reduces risk of IS.^3^ Whereas observational studies of a risk factor may be prone to confounding and other biases, the specific treatment assessed in a randomized trial may have effects beyond those produced by modifying the particular risk factor.

Genetic instruments that produce life-long exposure to a risk factor may help to determine whether associations with particular health outcomes are causal, and can also be used to elucidate the effects of biological mechanisms that are akin to therapeutic interventions. This ‘Mendelian randomization’ approach can avoid the difficulties in interpretation due to potential biases in observational studies and lack of specificity in randomized trials. However, to be able to detect or refute relevant effects, it requires the availability of genetic variants that produce sufficiently large effects on the risk factor (without material effects on other factors that may introduce bias) and of studies with sufficiently large numbers of the health outcome of interest. For example, for elucidating our understanding of LDL-C lowering therapies, many common variants in *HMGCR* are needed to mimic the biological mechanism by which statins exert their effects, as individual variants are associated with only small life-long differences in LDL-C levels. By contrast, single functional variants in the gene encoding proprotein convertase subtilisin/kexin type 9 (PCSK9) can be used to mimic pharmacological PCSK9-inihibition as they have large (about 10-fold greater) life-long effects on LDL-C levels.^4–7^

The present study assesses the association of *PCSK9* genetic variants with risk among more than 10 000 well-characterized IS cases, and compares its strength with the association for CHD risk.

## Methods

### Selection of *PCSK9* genetic variants

*PCSK9* variants were selected based on known variation in European ancestry groups, functional relevance and previously published associations. The primary focus was on the loss-of-function missense rs11591147 (R46L) polymorphism because, although relatively uncommon, it has a large effect on PCSK9 and LDL-C levels, and has been found to be strongly associated with CHD.^4^^,^^5^ In addition, three missense variants (rs562556 [V474I], rs505151[E670G], and rs11583680 [A53V]) and two non-coding variants (rs11206510 and rs2479409) were examined because, although associated with smaller effects on LDL-C and CHD, they are relatively common.^4^^,^^8–11^

### Study populations

Data were taken from three genome-wide meta-analyses: the Global Lipids Genetics Consortium (GLGC), CARDIoGRAMPlusC4D, and METASTROKE.^10^^,^^12^^,^^13^ The GLGC meta-analysis provides genome-wide associations of these *PCSK9* variants with LDL-C (and other lipids) in up to 188 577 participants, ranging from 77 417 participants for rs11591147 (R46L) to 172 970 participants for rs2479409.^10^ CARDIoGRAMPlusC4D provides genome-wide associations (from a 1000 Genomes imputation) with CHD risk in up to 60 801 CHD cases and 123 504 controls from 48 studies among individuals of predominantly European ancestry, with data available for rs11591147 [R46L] in 37 748 CHD cases and 97 202 controls from 31 studies.^12^

The METASTROKE collaboration provides genome-wide associations (from a 1000 Genomes imputation) with IS risk for all of these *PCSK9* variants in 10 307 IS cases and 19 326 controls of European ancestry from 12 studies.^13^ The majority of IS cases were recruited through acute stroke services or population studies, and were confirmed by brain imaging. Subtypes of IS (i.e. 1859 cardioembolic, 1817 large artery, and 1349 small vessel cases) were available in nine of the studies based on Trial of ORG 10172 in Acute Stroke Treatment (TOAST) classifications using clinical, imaging and risk factor data.^14^ Details of each contributing study are provided in the Supplementary materials, and additional information about the data collection and genetic data quality control procedures is reported elsewhere.^13^

### Statistical analyses

Per-allele effects of the selected *PCSK9* variants on LDL-C levels were extracted from the GLGC meta-analysis and converted from the published standard deviation units to mmol/L (based on 1 standard deviation being equivalent to 1.0083 mmol/L, as estimated by a pooled standard deviation from available studies contributing to GLGC).^10^ Per-allele log-odds effects of the variants on CHD risk were taken from the CARDIoGRAMPlusC4D genome-wide meta-analysis.^12^ In the METASTROKE meta-analysis, per-allele log-odds effects for each *PCSK9* variant were estimated in a fixed-effects meta-analysis for total IS and each IS subtype (large artery, cardioembolic, and small vessel disease).^13^ Estimates for disease phenotypes are given with respect to the LDL-C lowering allele. To account for multiple testing, an adjusted significance level of *P* < 0.008 was predefined for resulting associations. The effects of independent genetic variants were combined in a *PCSK9* genetic risk score using an inverse-variance approach, weighted by LDL-C estimates taken from the GLGC. Cochran’s Q statistic was used to test for heterogeneity. Analyses were performed in SAS v9.

## Results

### Impact of the rs11591147 (R46L) variant

**Figure 1:**
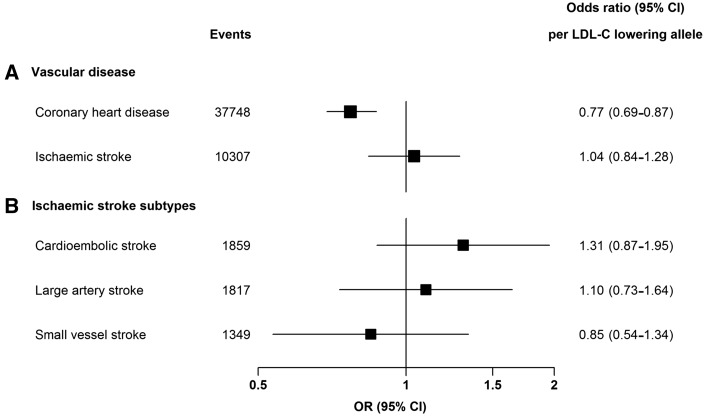
Associations of the *PCSK9* rs11591147 (R46L) variant (per LDL-C lowering allele) with risk of (*A*) coronary heart disease and ischaemic stroke and with (*B*) ischaemic stroke subtypes. Odds ratios (OR) and 95% confidence intervals (CI) are provided per T allele for each outcome. LDL-C, low-density lipoprotein cholesterol.

The low frequency (1.5%) rs11591147 (R46L) loss-of-function variant was associated with a 0.5 mmol/L [95% confidence interval (CI) 0.47*–*0.54, *P* *=* 9 × 10^−143^] lower LDL-C level per T allele. The LDL-lowering allele was associated with a 23% lower risk of CHD [odds ratio (OR) 0.77, 95% CI: 0.69*–*0.87, *P* *=* 7 × 10^−6^, *Figure 1*]. By contrast, the variant was not associated with IS risk (OR: 1.04, 95% CI: 0.84*–*1.28, *P* *=* 0.74, *Figure 1*), and there was no significant heterogeneity of the effect on IS between the contributing studies (*P* *=* 0.26). Overall, the association of rs11591147 (R46L) with IS risk was significantly different to that with CHD risk (*P* for heterogeneity *=* 0.02). These analyses had about 80% power at *P* < 0.008 (i.e. making allowance for multiple comparisons) to detect an effect for IS that was as large as that for CHD (although a smaller effect on IS cannot be excluded).

### Impact of additional *PCSK9* variants

The five additional *PCSK9* variants that were examined ranged in frequency from 14.1% to 96.5% for the LDL-lowering allele. Each of the additional *PCSK9* variants was significantly associated with LDL-C levels, with effects ranging from 0.09 mmol/L lower LDL-C per allele (95% CI: 0.07*–*0.11, *P* *=* 4 × 10^−17^) for rs505151 to 0.03 mmol/L lower LDL-C per allele (95% CI: 0.02*–*0.05, *P* *=* 1 × 10^−8^) for rs11583680 (*Table 1*). Although these variants appeared to be independent of rs11591147 (R46L) based on measures of *r*^2^, the D’ measure of genetic departure from independence was >0.6 for all except rs505151 (see Supplementary material online, *Table S1*). Consequently, the effects of each variant on risk were examined separately and a weighted genetic risk score was constructed including only the two independent variants (rs11591147 and rs505151).

**Table 1:** Effects of *PCSK9* variants on low-density lipoprotein cholesterol, coronary heart disease and ischaemic stroke

SNP	Effect/other allele	Effect allele frequency	LDL-C reduction		CHD		Ischaemic stroke		*P* Het
			mmol/L (95% CI)	*P*	OR (95% CI)	*P*-value	OR (95% CI)	*P*-value	(CHD vs. IS)
rs11591147	T/G	1.5%	0.50 (0.47–0.54)	9 × 10^−143^	0.77 (0.69–0.87)	7 × 10^−6^	1.04 (0.84–1.28)	0.74	0.02
rs505151	A/G	96.5%	0.09 (0.07–0.11)	4 × 10^−17^	0.96 (0.92–1.00)	0.07	1.00 (0.90–1.11)	0.98	0.50
rs11206510^a^	C/T	18.8%	0.08 (0.07–0.09)	2 × 10^−53^	0.93 (0.90–0.95)	2 × 10^−8^	1.01 (0.96–1.06)	0.84	0.01
rs2479409^a^	A/G	65.5%	0.06 (0.06–0.07)	3 × 10^−50^	0.97 (0.95–0.99)	0.01	1.00 (0.95–1.04)	0.86	0.31
rs562556^a^	G/A	18.3%	0.06 (0.05–0.08)	6 × 10^−21^	1.00 (0.98–1.03)	>0.99	1.06 (1.01–1.11)	0.03	0.05
rs11583680^a^	T/C	14.1%	0.03 (0.02–0.05)	1 × 10^−8^	0.97 (0.94–1.00)	0.03	1.00 (0.94–1.06)	0.89	0.40
Genetic score (per 1 mmol/L lower LDL-C)					0.60 (0.49–0.74)	1 × 10^−6^	1.07 (0.71–1.60)	0.76	0.01

LDL-C effect estimates are based on up to 172 970 individuals from the Global Lipids Consortium, CHD effect estimates are based on up to 60 801 CHD cases and 123 504 controls from the CARDIoGRAMplusC4D Consortium, and IS effect estimates are based on 10 307 IS cases and 19 326 controls from the METASTROKE Consortium. The genetic score is based on independent variants rs11591147 and rs505151. Estimates are given per effect allele unless otherwise stated.

LDL-C, low-density lipoprotein cholesterol; CHD, coronary heart disease; IS, ischaemic stroke; OR, odds ratio; CI, confidence interval; P Het, P for heterogeneity (1 degree of freedom) between CHD and IS.

a Variant is in linkage disequilibrium with rs11591147 (D’>0.6), for further information see Supplementary material online, *Table S1*.

**Figure 2:**
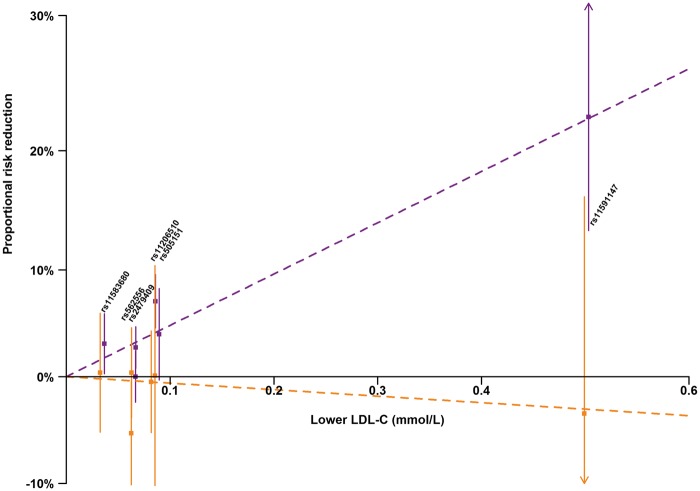
Proportional risk reduction of *PCSK9* variants on coronary heart disease and ischaemic stroke (per LDL-lowering allele) vs. absolute LDL-lowering effects. Effects on coronary heart disease (CHD) risk are shown in purple and effects on ischaemic stroke (IS) risk are shown in gold with dashed lines representing the estimated effects on risk based on the genetic risk score. Plotted points for CHD and IS are equally offset from the estimated effect on LDL-C to avoid overlap. LDL-C, low-density lipoprotein cholesterol.

*Figure 2* illustrates the weaker effect of each of these variants on IS risk than on CHD risk plotted against their effect on LDL-C level. Their effects on CHD were generally consistent with their effects on LDL-C, although there was some evidence of heterogeneity of the CHD effect sizes when they were scaled to the same LDL-C difference (*P* *=* 0.02). Consistent with the result for the rs11591147 (R46L) variant, there were no apparent associations of any of the other *PCSK9* variants with IS risk, and no significant heterogeneity between the effects of all six variants on IS risk when scaled to the same LDL-C difference (*P* *=* 0.56).

There were also no significant associations of rs11591147 (R46L) or any of the other *PCSK9* variants with cardioembolic, large artery or small vessel disease stroke (*Figure 1* and *Table 2*).

**Table 2:** Effects of *PCSK9* variants on ischaemic stroke subtypes

SNP	Effect/other allele	Effect allele frequency	Cardioembolic stroke		Large artery stroke		Small vessel disease	
			OR (95% CI)	*P*-value	OR (95% CI)	*P*-value	OR (95% CI)	*P*-value
rs11591147	T/G	1.5%	1.31 (0.87–1.95)	0.20	1.10 (0.73–1.64)	0.66	0.85 (0.54–1.34)	0.48
rs505151	A/G	96.5%	0.96 (0.78–1.16)	0.65	0.99 (0.80–1.21)	0.89	0.89 (0.71–1.11)	0.30
rs11206510^a^	C/T	18.8%	0.97 (0.88–1.06)	0.48	1.00 (0.91–1.10)	0.98	0.95 (0.85–1.05)	0.32
rs2479409^a^	A/G	65.5%	1.00 (0.92–1.09)	0.98	0.98 (0.90–1.07)	0.73	0.97 (0.89–1.07)	0.59
rs562556^a^	G/A	18.3%	1.06 (0.96–1.17)	0.24	1.12 (1.02–1.24)	0.02	0.99 (0.89–1.11)	0.88
rs11583680^a^	T/C	14.1%	0.95 (0.85–1.07)	0.42	0.97 (0.87–1.09)	0.64	0.94 (0.83–1.07)	0.35
Genetic score (per 1 mmol/L lower LDL-C)			1.51 (0.71–3.23)	0.28	1.16 (0.54–2.48)	0.71	0.64 (0.27–1.52)	0.31

Cardioembolic stroke effect estimates are based on 1859 cases, large artery stroke effect estimates are based on 1817 cases, and small vessel disease effect estimates are based on 1349 cases. The genetic score is based on independent variants rs11591147 and rs505151. Estimates are given per effect allele unless otherwise stated.

OR, odds ratio; CI, confidence interval.

a Variant is in linkage disequilibrium with rs11591147 (D’ >0.6), for further information see Supplementary material online, *Table S1*.

The *PCSK9* genetic risk score was associated with a 40% lower risk of CHD (OR: 0.60, 95% CI: 0.49*–*0.74, *P* *=* 1 × 10^−6^) but a non-significant effect on IS (OR: 1.07, 95% CI: 0.71*–*1.60, *P* *=* 0.76) per 1 mmol/L lower LDL-C, suggesting significant heterogeneity between the effects of *PCSK9* on CHD and on IS risk (*P* for heterogeneity *=* 0.01).

## Discussion

The present study is the first large-scale assessment of associations between *PCSK9* genetic variants and the risk of IS. *PCSK9* variants that confer life-long lower PCSK9 and LDL-C levels, and lower CHD risk, were not associated with risk of IS or IS subtypes. By contrast, in the recently reported FOURIER randomized controlled trial, PCSK9-inhibitor therapy that lowered LDL-C levels by about 1.5 mmol/L reduced the rates of both myocardial infarction and IS by about one-quarter over 2 years.^15^ In the SPIRE outcomes trials of PCSK9-inhibitor therapy which were prematurely terminated (and so involved smaller numbers of events), the rate of non-fatal stroke was also reduced by at least as much as for non-fatal myocardial infarction.^16^ These findings raise interesting questions about the cause of these apparent differences, as well as about the use of Mendelian randomization to predict the effects of novel therapeutic interventions.^17^

### Impact of *PCSK9* on cardiovascular risk factors

Mendelian randomization studies that assess the causal relevance of a risk factor for an outcome can avoid the reverse causation and confounding common in observational studies.^18^ However, pleiotropy in which a single locus directly influences multiple phenotypes can complicate their interpretation.^19^ The *PCSK9* genetic variants that were examined in the present study have previously been shown to have potential (*P* < 0.05) pleiotropic associations with non-LDL-C cardiovascular risk factors.^20^^,^^21^ For example, *PCSK9* variants associated with lower LDL-C have also been found to be associated with lower triglyceride and Lp(a) levels, as well as with differences in lipid metabolites and higher levels of HDL-C, fasting glucose, bodyweight, and rates of diabetes.^7^^,^^10^^,^^21–28^ However, many of these non-LDL-C effects of *PCSK9* variants are shared by therapeutic PCSK9-inhibition.^29–32^ Although the present study is not a direct test of the effects of LDL-C on IS risk (but, instead, is a test of PCSK9 levels on IS risk), the magnitude of association of rs11591147 (R46L) with CHD risk is broadly consistent with that predicted from Mendelian randomization studies of LDL-C and CHD.^33^^,^^34^ Hence, the CHD results are consistent with the effects of *PCSK9* being mediated chiefly through changes in LDL-C, with the combined impact of any other effects being only small or neutral.

Large-scale Mendelian randomization studies are needed that directly assess the relevance of specific, as well as varied, biological pathways that lower LDL-C in order to establish the broader causal relevance of life-long variation in LDL-C levels for IS risk. For example, variants in *HMGCR* could be used to examine the impact of the particular mechanism that is targeted by statin therapy, and its consequences on risk of IS and IS subtypes. However, to investigate the wider causal relevance of LDL-C levels (in contrast to PCSK9, or any other, drug target specifically), a genetic risk score which combines variants across the genome that reflect varied pathways (but are specifically associated with LDL-C levels) would be appropriate; such a score could also explain a greater proportion of the variance in LDL-C levels than any single variant and thereby provide greater statistical power.^35^ Assessment of the effects of a genetic score in studies involving sufficiently large numbers of disease cases is needed to determine reliably the effects of life-long differences in LDL-C on IS and IS subtypes.

### Impact of *PCSK9* on coronary heart disease and ischaemic stroke

The observed effects of *PCSK9* variants on CHD in the present study are broadly consistent with those reported previously.^4^^,^^6^^,^^33^^,^^34^ For example, Ference *et al*.^6^ reported a 28% (95% CI: 16% *–*39%) lower risk per rs11591147 T allele (albeit the CHD cases overlapped somewhat with those in the present study), and Benn *et al.*^4^ found a 30% (95% CI: 14*–*42%) lower risk of CHD in carriers of the LDL-lowering T allele. However, there did not appear to be an association of rs11591147 with stroke risk in the initial studies of *PCSK9* among individuals of African American or European ancestry, although the number of strokes observed was small.^4^^,^^5^ In other candidate-gene studies, the observed associations of *PCSK9* variants with IS risk have been inconsistent.^36–39^ Studies of other functional *PCSK9* variants that produce large effects on PCSK9 and LDL-C levels (e.g. mutations in Y142X or C679X associated with life-long differences in LDL-C levels of about 1 mmol/L) have not detected associations with stroke.^5^^,^^40^ However, such loss-of-function mutations are rare (about 2% frequency in individuals of African ancestry and far lower frequency in individuals of European ancestry) and the existing studies have involved only about 200 stroke cases.

In a recently reported study involving 3675 cases of stroke of any aetiology, the association of a *PCSK9* genetic risk score (that excluded the rs11591147 variant, which has a considerably larger effect on LDL-C levels than the variants considered) with the risk of stroke was also weaker (OR: 0.96, 95% CI: 0.90*–*1.01) than was the association with non-fatal myocardial infarction (OR: 0.89, 95% CI: 0.85*–*0.94), when comparing participants with a *PCSK9* score above and below the median.^7^ That observation is less robust than the present results since it was based on about one-third as many strokes, and was not restricted to strokes of ischaemic origin. Stroke research has often been complicated by diagnostic limitations and phenotypic heterogeneity (i.e. combining strokes of ischaemic and haemorrhagic aetiology), as well as by an inability to distinguish IS subtypes. However, in the present study, the majority of IS cases were confirmed and subtyped based on brain imaging and/or a specialist neurologist examination, reducing the potential impact of misclassification. Nonetheless, much larger studies are needed for reliable assessment of genetic effects on aetiologically distinct IS subtypes and to assess any heterogeneity between them, which may elucidate our understanding of the relationship between PCSK9, LDL-C, and IS further.

Differences in the impact of *PCSK9* on the risks of IS and CHD in the present study may represent biological differences, such as relative differences in the contribution of lipids, as well as phenotypic and design artefacts. Exploration of canonical pathways has suggested that genetic determinants of CHD are linked to lipid metabolism pathways whereas, by contrast, those for IS are linked to natural killer cell signalling rather than to lipid pathways.^9^^,^^13^ IS also involves phenotypic heterogeneity, with different biological drivers for cardioembolic, large artery and small vessel disease, by contrast to the more homogenous CHD phenotype. Genetic associations may be stronger for disease cases that occur at a younger age which, in principle, could be a confounding factor in the present comparison between *PCSK9* associations for CHD and IS derived from different studies. However, the age range for CHD and IS cases in the studies contributing to these analyses was similar, so the impact of age-related confounding is likely to be limited. Studies involving larger numbers of CHD and IS cases within the same study may well help to elucidate the role of such factors in Mendelian randomization studies.

### Mendelian randomization vs. randomized trials

ESC/EAS guidelines recommend consideration of PCSK9-inhibitor therapy in very high-risk patients with persistently high LDL-C levels despite treatment with maximal tolerated statin therapy plus ezetimibe.^41^ Based on the present study, PCSK9-inhibitor therapy would have been predicted to have a significantly weaker, if any, effect on risk of IS than on risk of CHD. Recent randomized trials have shown comparable effects of PCSK9-inhibition on IS and myocardial infarction, although the lower confidence limits are also consistent with a weaker effect on IS than on myocardial infarction (8% vs. 18% risk reduction, respectively).^15^ The FOURIER and SPIRE trials recruited populations at high risk of atherosclerotic events, with all participants in FOURIER having clinically evident atherosclerotic cardiovascular disease (including 80% with a history of myocardial infarction) and almost 85% of participants in SPIRE-1 and SPIRE-2 having had a previous cardiovascular event (and the remaining 15% being a high risk primary prevention cohort).^15^^,^^16^ By contrast, the risk factor and comorbidity profiles of individuals included in the METASTROKE studies represent a population that is less enriched for atherosclerotic disease. Consequently, by comparison with the trials, a larger proportion of IS events in METASTROKE may have been driven by risk factors that are not amenable to lipid modification (such as hypertension and atrial fibrillation), resulting in attenuation of the genetic associations. Further large studies are needed with sufficient statistical power to consider the impact of population characteristics (such as risk of disease) on the results of Mendelian randomization studies.

**Take home figure:**
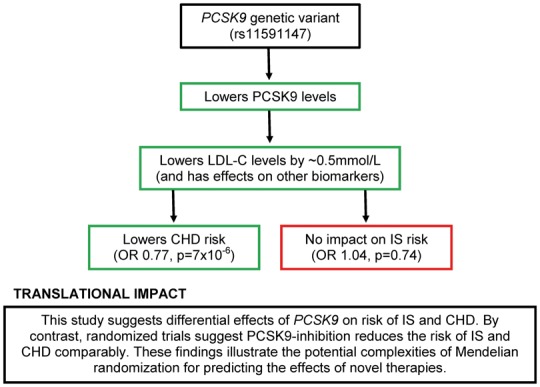
Overview of the impact of *PCSK9* variants on CHD and IS.

## Conclusion

*PCSK9* genetic variants that produce life-long lower levels of LDL-C, and that are associated with lower risk of CHD, appear to have a significantly weaker (if any) effect on the risk of IS. By contrast, PCSK9-inhibitor therapy (as with statin therapy and ezetimibe) has been shown to reduce the risk of CHD and stroke comparably. These findings illustrate potential limitations with the use of Mendelian randomization to predict the effects of novel therapeutic interventions on different health outcomes.^17^

## Supplementary material

Supplementary material is available at *European Heart Journal* online.

## Supplementary Material

Supplementary Data
