# Supplementary material for: Differential effects of PCSK9 variants on risk of coronary disease and ischaemic stroke
Source: Eur Heart J. 2017 Jul 17;39(5):354–9. doi: 10.1093/eurheartj/ehx373 (PMC5837489; doi:10.1093/eurheartj/ehx373)
Supplement: Supplementary Data [file ehx373_hopewell_pcsk9_supplementary.pdf]

**Supplementary Data**

**Contents**

METASTROKE Collaboration individual study descriptions Page 2

METASTROKE Collaboration study level acknowledgements Page 7

Table S1: Linkage disequilibrium information for selected *PCSK9* variants Page 9

**METASTROKE Collaboration individual study descriptions**

Additional information is also provided in Malik et al. Low frequency and common variation in ischaemic stroke: the METASTROKE collaboration. *Neurology* 2016; 86 (13): 1217-1226

**Australian Stroke Genetics Collaborative (ASGC)**

ASGC stroke cases comprised European-ancestry stroke patients admitted to four clinical centres across Australia (The Neurosciences Department at Gosford Hospital, Gosford, New South Wales (NSW); the Neurology Department at John Hunter Hospital, Newcastle, NSW; The Queen Elizabeth Hospital, Adelaide; and the Royal Perth Hospital, Perth) between 2003 and 2008. Stroke was defined by WHO criteria as a sudden focal neurologic deficit of vascular origin, lasting more than 24 hours and confirmed by imaging such as computerised tomography (CT) and/or magnetic resonance imaging (MRI) brain scan. Other investigative tests such as electrocardiogram, carotid doppler and transoesophageal echocardiogram were conducted to define IS mechanism as clinically appropriate. Cases were excluded from participation if aged <18 years, diagnosed with haemorrhagic stroke or transient ischemic attack rather than IS, or were unable to undergo baseline brain imaging. Based on these criteria, a total of 1230 IS cases were included in the current study. IS subtypes were assigned using TOAST criteria, based on clinical, imaging and risk factor data. ASGC controls were participants in the Hunter Community Study (HCS), a population-based cohort of individuals aged 55-85 years, predominantly of European Caucasian ancestry and residing in the Hunter Region, NSW, Australia. Detailed recruitment methods for the HCS have been previously described. Briefly, participants were randomly selected from the NSW State electoral roll and contacted by mail between 2004 and 2007. Consenting participants completed five detailed self-report questionnaires and attended the HCS data collection centre, at which time a series of clinical measures were obtained. A total of 1280 HCS participants were genotyped for the current study. All study participants gave informed consent for participation in genetic studies. Approval for the individual studies was obtained from relevant institutional ethics committees.

**Bio-Repository of DNA in Stroke (BRAINS)**

The Bio-Repository of DNA in Stroke (BRAINS) is an international study recruiting highly phenotyped patients with stroke. For the purposes of the current work all patients were Caucasians. Diagnosis of stroke was confirmed using positive imaging (MRI or CT) and ischemic stroke subtypes were assigned using TOAST criteria, based on clinical, imaging and risk factor data. Controls were European-Ancestry, stroke-free participants from the shared WTCCC controls, a prospectively collected cohort of individuals born in 1958 (1958 Birth Cohort). The cohort has been described in detail elsewhere.

**The Genetics of Early Onset Stroke (GEOS) Study, Baltimore, USA**

GEOS is a population-based case-control study designed to identify genes associated with early onset stroke in patients with first-ever ischemic stroke aged 15-49 years from the greater Baltimore- Washington area between 1992 and 2008. Only patients of European descent are included in this meta-analysis. Cases were identified through discharge surveillance from 59 participating hospitals and direct physician referral from a defined geographic region.

Abstracted medical records were reviewed and adjudicated for ischemic stroke subtype by two neurologists, with discrepancies resolved by a third neurologist. Controls with no history of ischemic stroke were identified through random digit dialing and were frequency-matched to cases based on sex, age, geographic location and, during the later study periods, ethnicity.

**Heart Protection Study (HPS)**

The Heart Protection Study (HPS) was a large randomized trial involving individuals at increased risk of vascular events. Between 1994-1997 20,536 men and women aged 40-80 years were recruited from 69 collaborating hospitals in the United Kingdom (with ethics committee approval). Participants were eligible for inclusion provided they had non-fasting blood total cholesterol concentrations of at least 135 mg/dL (3.5 mmol/L) and either a previous diagnosis of coronary disease, ischemic stroke, other occlusive disease of non-coronary arteries, diabetes mellitus, or (if were men 65 years or older) treated hypertension. None of them was on statin therapy. At the initial screening visit, all participants provided written consent and began a "runin" phase involving 4 weeks of placebo followed by 4 to 6 weeks of 40 mg simvastatin daily, after which compliant and eligible individuals were randomly allocated 40 mg simvastatin daily or matching placebo for approximately 5 years. Individuals entering HPS with a clinical diagnosis of ischemic stroke were used as cases in the METASTROKE study. Individuals entering HPS with pre-existing diabetes but no history of cerebrovascular disease, coronary heart disease or peripheral vascular disease were used as controls.

**The Ischemic Stroke Genetics Study (ISGS)/ Siblings With Ischemic Stroke Study (SWISS)**

The Siblings with Ischemic Stroke Study (SWISS) is a multicenter affected sibling pair study enrolling probands with ischemic stroke at 66 US medical centers and 4 Canadian medical centers. All probands are adult men and women over the age of 18 years diagnosed with ischemic stroke confirmed by a study neurologist on the basis of history, physical examination and CT or MR imaging of the brain. Additionally all probands were required to have at least one living sibling with a history of stroke. Siblings were enrolled using proband-initiated contact or direct contact when permitted by Institutional Review Boards. Clinical exclusion criteria mirrored that in ISGS. Concordant siblings had their diagnosis of ischemic stroke confirmed by review of medical records by a central vascular neurology committee. Subtype diagnoses were assigned to the index strokes of probands and concordant siblings according to TOAST criteria. Readily available US controls were utilized, including stroke-free participants from the Baltimore Longitudinal Study of Aging and the National Institute of Neurological Diseases and Stroke neurologically normal control series taken from the Coriell Cell Repositories. All controls had been previously genotyped and described in detail elsewhere.

The Ischemic Stroke Genetic Study (ISGS) is a multicenter study where inpatient cases were recruited from five United States academic medical centers. Cases are adult men and women over the age of 18 years diagnosed with first-ever ischemic stroke confirmed by a study neurologist on the basis of history, physical examination and CT or MR imaging of the brain who were enrolled within 30 days of onset of stroke symptoms. Cases exclusion criteria include: a mechanical aortic or mitral valve at the time of the index ischemic stroke, central nervous system vasculitis, bacterial endocarditis, cerebral autosomal dominant arteriopathy with subcortical infarcts and leukoencephalopathy (CADASIL), Fabry disease, homocystinuria, mitochondrial encephalopathy with

lactic acidosis and stroke-like episodes (MELAS), or sickle cell anemia. Stroke severity at enrollment was assessed using the NIH Stroke Scale with the diagnostic evaluation including head CT (95%) or MRI (83%), electrocardiography (92%), cervical arterial imaging (86%), and echocardiography (74%). Medical records from all cases were centrally reviewed by a vascular neurology committee and assigned ischemic stroke subtype diagnoses according to criteria from the Trial of ORG10172 (TOAST), the Oxfordshire Community Stroke Project, and the BaltimoreWashington Young Stroke Study.

**The MGH Genes Affecting Stroke Risk and Outcome Study (MGH-GASROS)**
Cases were all consecutive patients aged 18 years presenting with ischemic stroke and admitted to the Massachusetts General Hospital (MGH) Stroke Unit through the Emergency Department, or evaluated in the MGH Neurology outpatient clinics, as well as on the inpatient Medical and Vascular Surgical services from January 2003 to July 2008. Only patients of European ancestry (confirmed by principal component analysis using genome-wide SNP data) were included in the present analysis. Ischemic stroke was defined as either (1) a radiographically proven (head CT or MRI) infarct associated with the appropriate clinical stroke syndrome, or (2) a fixed neurological deficit persisting more than 24 hours, consistent with a vascular pattern of involvement and without radiographic evidence of demyelinating disease, or other non-vascular structural disease. Patients with specific vascular disorders (vasculitis, subacute bacterial endocarditis, fibromuscular dysplasia, vasospasm) were excluded from the study. All subjects were evaluated by a neurologist upon presentation and provided informed consent. Clinical and laboratory data were collected during the admission for qualifying ischemic stroke event. Diagnostic work-up included: head CT (100%), brain MRI (90%), cervical and intracranial vessel imaging using CTA or MRA (75%), carotid and/or transcranial ultrasound (24%), echocardiography (86%), and Holter monitoring (16%). Controls were recruited among the stroke-free adults presenting to the MGH outpatient clinics and matched with the stroke cases on the basis of age, sex and ancestry information obtained from principal component analysis of GWAS data.

**Milano**
This study includes consecutive Italian patients referred to Besta Institute from 2000 to 2009 with stroke and included in the Besta Cerebrovascular Diseases Registry (CEDIR). Ischemic stroke cases, first ever or recurrent, confirmed on brain imaging, were selected for this study. All cases were of self-reported Caucasian ancestry and had clinically relevant diagnostic workup performed. All cases were phenotyped by an experienced stroke neurologist according to TOAST criteria, based on relevant clinical imaging and available information on cardiovascular risk factors. Controls are Italian individuals enrolled within the PROCARDIS Study, with no personal or sibling history of coronary heart disease before age 66 years.

**Wellcome Trust Case-Control Consortium 2 (WTCCC2)**
The WTCCC2 samples were genotyped as part of the WTCCC 2 ischemic stroke study. Stroke cases included samples recruited by investigators at St. George's University London (SGUL) and University of Oxford in the UK and the Department of Neurology, Klinikum Großhadern, LudwigMaximilians-University, Munich. The SGUL collection comprised 1224 ischemic stroke samples from a hospital based setting. All cases were of self -reported Caucasian ancestry. Ischemic stroke subtypes were determined according to TOAST criteria based on relevant clinical imaging and available information on

cardiovascular risk factors. The University of Oxford collection comprised 896 ischemic stroke cases, consecutively collected as part of the Oxford vascular study (OXVASC). Cases were of self- reported Caucasian ancestry, and ischemic stroke subtypes were determined according to TOAST criteria based on relevant clinical imaging. The University of Edinburgh collection comprised 727 ischaemic stroke cases, consecutively collected as part of the Edinburgh Stroke Study. Cases were of self-reported Caucasian ancestry, with ischaemic stroke subtypes determined according to TOAST criteria based on relevant clinical and imaging data. The Munich samples included 1383 ischemic stroke cases. Cases were consecutive European Caucasians recruited from a single dedicated Stroke Unit at the Department of Neurology, Klinikum Großhadern, Ludwig-Maximilians-University, Munich. Ischemic stroke subtypes were determined according to TOAST criteria based on relevant clinical and imaging data. Controls for the UK samples were drawn from shared WTCCC controls obtained from the 1958 Birth Cohort. This is a prospectively collected cohort of individuals born in 1958 ), and ascertained as part of the national child development study. Data from this cohort are available as a common control set for a number of genetic and epidemiological studies. For the German samples controls were Caucasians of German origin participating into the population KORAgen study. This survey represents a gender- and age stratified random sample of all German residents of the Augsburg area and consists of individuals 25 to 74 years of age, with about 300 subjects for each 10-year increment. All controls were free of a history of stroke or transient ischemic attack.

**VISP**

The VISP trial (P.I. James Toole, MD, Wake Forest University School of Medicine (WFU); R01NS34447) was a multi-center, double-blind, randomized, controlled clinical trial that enrolled patients aged 35 or older with Homocysteine levels above the 25th percentile at screening and a non-disabling cerebral infarction (NDCI) within 120 days of randomization. NDCI was defined as an ischemic brain infarction not due to embolism from a cardiac source, characterized by the sudden onset of a neurological deficit. The deficit must have persisted for at least 24 hours, or if not, an infarction in the part of the brain corresponding to the symptoms must have been demonstrated by CT or MRI imaging. The trial was designed to determine if daily intake of a multivitamin tablet with high dose folic acid, vitamin B6 and vitamin B12 reduced recurrent cerebral infarction (1° endpoint), and nonfatal myocardial infarction (MI) or mortality (2° endpoints). Subjects were randomly assigned to receive daily doses of the high-dose formulation (n=1,827), containing 25mg pyridoxine (B6), 0.4mg cobalamin (B12), and 2.5mg folic acid; or the low-dose formulation (n=1,853), containing 200μg pyridoxine, 6μg cobalamin and 20μg folic acid. Enrollment in VISP began in August 1997, and was completed in December 2001, with 3,680 participants enrolled, from 55 clinic sites across the US and Canada and one site in Scotland. Control data for comparison with VISP stroke cases were obtained through the database of genotypes and phenotypes (dbGAP) High Density SNP Association Analysis of Melanoma: Case-Control and Outcomes Investigation (phs000187.v1.p1; R01CA100264, 3P50CA093459, 5P50CA097007, 5R01ES011740, 5R01CA133996, HHSN268200782096C; PIs Christopher Amos, Qingyi Wei, Jeffrey E. Lee).

**WHI**

The WHI Hormone Trial HT (WHI-HT) consisted of two separate clinical trials in postmenopausal women ages 50 to 79 years at baseline—a trial of combined estrogen and progestin (Estrogen plus Progestin or E+P) in women who had an intact uterus at baseline (n=16,608) and a trial of estrogen (Estrogen Alone or E-Alone) in women who had a prior hysterectomy at baseline (n=10,739). Postmenopausal women who gave written informed consent were

enrolled in the WHI at 40 clinical centers in the United States. Exclusions for safety reasons included prior diagnosis of breast cancer or other cancers within the past 10 years (except nonmelanoma skin cancer). Women with systolic blood pressure (SBP) of 200 mm Hg or higher or diastolic blood pressure (DBP) of 105 mm Hg or higher were advised to see their physician within a specified period depending on blood pressure level and were temporarily excluded from the clinical trials until their blood pressure was determined to be under control. Stroke diagnosis requiring and/or occurring during hospitalization was based on rapid onset of a neurological deficit attributable to an obstruction or rupture of an arterial vessel system. Hospitalized incident stroke events were identified by semiannual questionnaires and adjudicated following medical record review, which occurred both locally and centrally. Ischemic strokes were further classified by the central neurologist adjudicators according to the Oxfordshire and Trial of Org 10172 Acute Stroke Trial (TOAST) criteria to examine stroke subtypes. The TOAST classification focuses on the presumed underlying stroke mechanism and requires detailed investigations (such as brain computed tomography, magnetic resonance imaging, angiography, carotid ultrasound, and echocardiography).

**METASTROKE Collaboration study level acknowledgements**

***Australian Stroke Genetics Collaboration (ASGC)*** Australian population control data were derived from the Hunter Community Study. We also thank the University of Newcastle for funding and the men and women of the Hunter region who participated in this study. This research was funded by grants from the Australian National and Medical Health Research Council (NHMRC Project Grant ID: 569257), the Australian National Heart Foundation (NHF Project Grant ID: G 04S 1623), the University of Newcastle, the Gladys M Brawn Fellowship scheme, and the Vincent Fairfax Family Foundation in Australia. Elizabeth G Holliday was supported by a Fellowship from the National Heart Foundation and National Stroke Foundation of Australia (ID: 100071). ***Bio-Repository of DNA in Stroke (BRAINS)*** is partly funded by a Senior Fellowship from the Department of Health (UK) to P Sharma, the Henry Smith Charity and the UK-India Education Research Institutive (UKIERI) from the British Council. ***Genes Affecting Stroke Risk and Outcome Study (GASROS)*** was supported by NINDS (U01 NS069208), the American Heart Association/Bugher Foundation Centers for Stroke Prevention Research 0775010N, the NIH and NHLBI's STAMPEED genomics research program (R01 HL087676), and a grant from the National Center for Research Resources. The Broad Institute Center for Genotyping and Analysis is supported by grant U54 RR020278 from the National Center for Research resources. ***Genetics of Early Onset Stroke (GEOS) Study***, Baltimore, USA was supported by the NIH Genes, Environment and Health Initiative (GEI) Grant U01 HG004436, as part of the GENEVA consortium under GEI, with additional support provided by the Mid-Atlantic Nutrition and Obesity Research Center (P30 DK072488), and the Office of Research and Development, Medical Research Service, and the Baltimore Geriatrics Research, Education, and Clinical Center of the Department of Veterans Affairs. Genotyping services were provided by the Johns Hopkins University Center for Inherited Disease Research (CIDR), which is fully funded through a federal contract from the NIH to the Johns Hopkins University (contract number HHSN268200782096C). Assistance with data cleaning was provided by the GENEVA Coordinating Center (U01 HG 004446; PI Bruce S Weir). Study recruitment and assembly of datasets were supported by a Cooperative Agreement with the Division of Adult and Community Health, Centers for Disease Control and Prevention and by grants from NINDS and the NIH Office of Research on Women's Health (R01 NS45012, U01 NS069208-01). ***Heart Protection Study (HPS)*** (ISRCTN48489393) was supported by the UK Medical Research Council (MRC), British Heart Foundation, Merck and Co (manufacturers of simvastatin), and Roche Vitamins Ltd (manufacturers of vitamins). Genotyping was supported by a grant to Oxford University and CNG from Merck and Co. Jemma C Hopewell acknowledges support from the British Heart Foundation (FS/14/55/30806). ***Ischemic Stroke Genetics Study (ISGS)/Siblings With Ischemic Stroke Study (SWISS)*** was supported in part by the Intramural Research Program of the NIA, NIH project Z01 AG-000954-06. ISGS/SWISS used samples and clinical data from the NIH-NINDS Human Genetics Resource Center DNA and Cell Line Repository (http://ccr.coriell.org/ninds), human subjects protocol numbers 2003-081 and 2004-147. ISGS/SWISS used stroke-free participants from the Baltimore Longitudinal Study of Aging (BLSA) as controls. The inclusion of BLSA samples was supported in part by the Intramural Research Program of the NIA, NIH project Z01 AG-000015-50, human subjects protocol number 2003-078. The ISGS study was funded by NIH-NINDS Grant R01 NS-42733 (J F Meschia). The SWISS study was funded by NIH-NINDS Grant R01 NS-39987 (J F Meschia). This study used the high-performance computational capabilities of the Biowulf Linux cluster at the NIH (http://biowulf.nih.gov). ***Milano - Besta Stroke Register Collection*** and genotyping of the Milan cases within CEDIR were supported by Annual Research Funding of the Italian Ministry of Health (Grant Numbers: RC 2007/LR6, RC 2008/LR6; RC 2009/LR8; RC 2010/LR8). FP6 LSHM-CT-2007-037273 for the PROCARDIS control samples. ***Wellcome Trust Case-Control***

***Consortium 2 (WTCCC2)*** was principally funded by the Wellcome Trust, as part of the Wellcome Trust Case Control Consortium 2 project (085475/B/08/Z and 085475/Z/08/Z and WT084724MA). The Stroke Association provided additional support for collection of some of the St George's, London cases. The Oxford cases were collected as part of the ***Oxford Vascular Study*** which is funded by the MRC, Stroke Association, Dunhill Medical Trust, National Institute of Health Research (NIHR) and the NIHR Biomedical Research Centre, Oxford. The ***Edinburgh Stroke Study*** was supported by the Wellcome Trust (clinician scientist award to C Sudlow), and the Binks Trust. Sample processing occurred in the Genetics Core Laboratory of the Wellcome Trust Clinical Research Facility, Western General Hospital, Edinburgh. Much of the neuroimaging occurred in the Scottish Funding Council Brain Imaging Research Centre (www.sbirc.ed.ac.uk), Division of Clinical Neurosciences, University of Edinburgh, a core area of the Wellcome Trust Clinical Research Facility and part of the SINAPSE (Scottish Imaging Network—A Platform for Scientific Excellence) collaboration (www.sinapse.ac.uk), funded by the Scottish Funding Council and the Chief Scientist Office. Collection of the Munich cases and data analysis was supported by the Vascular Dementia Research Foundation. ***Vitamin Intervention Stroke Prevention (VISP)*** The GWAS component of the VISP study was supported by the United States National Human Genome Research Institute (NHGRI), Grant U01 HG005160 (PI Michèle Sale & Bradford Worrall), as part of the Genomics and Randomized Trials Network (GARNET). Genotyping services were provided by the Johns Hopkins University Center for Inherited Disease Research (CIDR), which is fully funded through a federal contract from the NIH to the Johns Hopkins University. Assistance with data cleaning was provided by the GARNET Coordinating Center (U01 HG005157; PI Bruce S Weir). Study recruitment and collection of datasets for the VISP clinical trial were supported by an investigator-initiated research grant (R01 NS34447; PI James Toole) from the United States Public Health Service, NINDS, Bethesda, Maryland. Control data for comparison with VISP stroke cases were from the dbGaP study High Density SNP Association Analysis of Melanoma: Case-Control and Outcomes Investigation (phs000187.v1.p1; R01CA100264, 3P50CA093459, 5P50CA097007, 5R01ES011740, 5R01CA133996, HHSN268200782096C; PIs Christopher Amos, Qingyi Wei, Jeffrey E. Lee). ***Women's Health Initiative (WHI )*** Funding support for WHI-GARNET was provided through the NHGRI GARNET (Grant Number U01 HG005152). Assistance with phenotype harmonisation and genotype cleaning, as well as with general study coordination, was provided by the GARNET Coordinating Center (U01 HG005157). Funding support for genotyping, which was performed at the Broad Institute of MIT and Harvard, was provided by the NIH Genes, Environment, and Health Initiative (GEI; U01 HG004424).

**Table S1: Linkage disequilibrium information for selected *PCSK9* variants**

Information from 1000 Genomes CEU reference population, $r^2$ is given in standard type face and D' is given in italics

| | **rs11591147** | **rs505151** | **rs11206510** | **rs2479409** | **rs562556** | **rs11583680** |
|---|---|---|---|---|---|---|
| **rs11591147** | 1.00 | | | | | |
| | *1.00* | | | | | |
| **rs505151** | 0.00 | 1.00 | | | | |
| | *0.00* | *1.00* | | | | |
| **rs11206510** | 0.04 | 0.01 | 1.00 | | | |
| | *0.67* | *1.00* | *1.00* | | | |
| **rs2479409** | 0.01 | 0.00 | 0.04 | 1.00 | | |
| | *1.00* | *0.21* | *0.64* | *1.00* | | |
| **rs562556** | 0.03 | 0.01 | 0.01 | 0.03 | 1.00 | |
| | *0.64* | *1.00* | *0.13* | *0.52* | *1.00* | |
| **rs11583680** | 0.00 | 0.01 | 0.25 | 0.06 | 0.05 | 1.00 |
| | *0.94* | *1.00* | *0.56* | *0.94* | *0.28* | *1.00* |
